# Cuproptosis related genes in immune infiltration and treatment of osteoporosis by bioinformatic analysis and machine learning methods

**DOI:** 10.3389/fphys.2025.1605473

**Published:** 2025-09-04

**Authors:** Haiyang Wu, Junhao Wu, Guowei Wen

**Affiliations:** Shanghai Second People’s Hospital, Shanghai, China

**Keywords:** cuproptosis, Osteoporosis, cell death, inflammation, machine learning

## Abstract

Cuproptosis, a copper-dependent form of cell death, has been implicated in immune function and osteoporosis. However, the specific roles of cuproptosis-related genes (CRGs) in osteoporosis remain unclear. The differentially expressed CRGs from the Gene Expression Omnibus datasets of persons with osteoporosis and healthy individuals were categorized using R software tools in this study. Following that, the CIBERSORT algorithm and the GSVA technique were used to investigate the relationships between the different clusters and immune infiltration characteristics. Based on four machine learning techniques (Random Forest, Support Vector Machine, XGBoost, and Generalized Linear Model), Support Vector Machine and WGCNA analysis was carried out to identify the main genes linked to cuproptosis in the pathological course of osteoporosis. Subsequently, a model was built using the core genes related to cuproptosis to forecast the disease and identify potential treatment targets. The model was validated using an external dataset. In the end, a nomogram and calibration curve were created to improve this model’s clinical applicability. Additionally, to investigate the possible biological roles of the core genes related to cuproptosis, we enriched them along several pathways. This study represents the first identification of key CRGs and core genes associated with cuproptosis in osteoporosis patients, findings that will facilitate the development of novel therapeutic strategies.

## Introduction

Osteoporosis, a systemic skeletal disease characterized by reduced bone mass, microarchitectural deterioration of bone tissue, increased bone fragility, and elevated fracture risk (Ensrud and Crandall, 2024). In the US during 2017–2018, the age-adjusted prevalence of osteoporosis among adults aged ≥50 years was 12.6% (Sarafrazi et al., 2021). Interventions for prevention and treatment include hormone replacement therapy, bisphosphonates, Denosumab, Raloxifene, Parathyriod homone peptides, Romosozumab, calcium and vitamin D, herbs and exercise (Liu et al., 2017; Stevenson and medical advisory council of the British Menopause, 2023). Despite these interventions, approximately two million incident osteoporotic fractures occur annually in the US, incurring an estimated cost of $17 billion (Sarafrazi et al., 2021). Adverse fracture-related impacts include functional impairment, chronic pain, lower quality of life, loss of independence, and increased mortality, especially after hip and clinical vertebral fractures (Singer, 2021). Alarmingly, osteoporosis management is frequently neglected in older patients following fracture events (Curtis et al., 2020).

Between 50 and 120 mg of copper are found in our bodies, with around two-thirds of that amount found in muscles and bones (Turnlund et al., 1998). Copper ions play critical roles in numerous biological processes, including kinase activity regulation, organellar redox balance maintenance, and gene expression modulation (Henriksen and Arnesen, 2023). However, overexposure to copper can cause cell death via processes such mitochondrial malfunction, proteasome suppression, and the buildup of reactive oxygen species (ROS) (Chen et al., 2023). This copper-dependent cell death, termed “cuproptosis,” has been implicated in osteoporosis pathogenesis (Chaudhri et al., 2009; Mahdavi-Roshan et al., 2015; Qu et al., 2018). It involves the tricarboxylic acid cycle’s binding of copper ions to fatty-acylated proteins, which causes proteotoxic stress, an inflammatory response, and cell death (Tsvetkov et al., 2022; Li et al., 2023).

However, the precise mechanistic role of cuproptosis in osteoporosis and its connection to the disease pathology remain complex and incompletely understood. Cuproptosis exhibits significant interplay with other regulated cell death pathways, including autophagy, apoptosis, pyroptosis, and ferroptosis (Liu M. et al., 2023; Xue et al., 2023). For example, pro-apoptotic mitogen-activated protein kinase pathways can be triggered by copper-induced ROS, ultimately resulting in apoptosis (Chen et al., 2009). Similarly, macrophage pyroptosis, an inflammatory kind of cell death, can be brought on by copper-mediated activation of the NLRP3 inflammasome pathway (Qiao et al., 2024). Moreover, copper overload has been observed to increase free iron pools and disrupt mitochondrial homeostasis, suggesting a mechanistic link to ferroptosis (Liu and Chen, 2024).

The dual function of copper in cellular processes and its potential toxicity when homeostasis is disturbed have been brought to light by recent investigations (Chen et al., 2023). Consuming copper may lower the incidence of osteoporosis and be favorably correlated with bone mineral density (Mir et al., 2007). For instance, correlations between copper consumption and bone mineral density were discovered in research using data from the National Health and Nutrition Examination Survey, indicating that copper may have preventive effects on bone health​​ (Fan et al., 2022). Copper supplementation has also demonstrated anti-inflammatory and anti-arthritic properties, potentially preserving articular cartilage and modulating immune responses (Liu Y. et al., 2022). Conversely, copper poisoning is a well-known concern that may result in a number of health issues, such as disruptions in the enzymes responsible for mitochondrial metabolism and cell death (Zhang and Burke, 2023)​​​​. To get the benefits of copper, it's critical to strike a balance between consuming enough of it without going beyond, as this may be hazardous (Ge et al., 2022). To properly comprehend the role of copper in bone health and osteoporosis, more research is necessary, as the results of this study do not align with those of the other studies.

Consequently, it is critical to identify the core cuproptosis-related genes (CRGs) that regulate the biological process and the mechanism of cuproptosis in the pathology of osteoporosis. We carried out this bioinformatic study with the intention of offering a fresh perspective to enhance comprehension of the underlying biological process and search for novel treatment strategies by searching the relevant genes. To our knowledge, this is the first study to use bioinformatic and machine learning techniques to investigate cuproptosis in osteoporosis.

## Methods

Whole flow: Data collection and differential expressed CRGs identification →Immune infiltration analysis →Subtype clustering →Hub Gene selection →Model construction and validation →Annotation and drug prediction.

### GEO data sources and differential expressed CRGs acquirement

Gene expression datasets were downloaded from the National Center for Biotechnology Information (NCBI) Gene Expression Omnibus (GEO) database. The selection criteria are as follows: diagnosed by a professional institution according to the bone mineral density; no restriction of the osteoporosis type, sex, age, and race. The analyzed dataset (GSE56815) and validation datasets (GSE7429 and GSE35957) were selected based on their relevance to osteoporosis and the availability of comprehensive gene expression profiles (Xiao et al., 2008; Benisch et al., 2012; Zhou et al., 2018). The GSE56815 dataset involved 40 osteoporosis patients with hip Z-score < −0.52 and 40 controls with hip Z-score >0.84 on the platform of GPL96. This trial was approved by the Institutional Review Boards of University of Missouri Kansas City and Tulane University. Written informed consent was obtained from all participants before inclusion. The GSE7429 dataset involved 10 osteoporosis cases with a spine or hip Z-score < −0.84 and 10 controls with a spine or hip Z-score >0.84 on the platform of GPL96. Institutional Review Board of Creighton University in Omaha approved this study, and all the subjects signed informed-consent documents before participating in the trial. The GSE35957 dataset included five controls and five osteoporosis patients on the platform of GPL570. Bone material was used under agreement of the local Ethics Committee of the Medical Faculty of the University of Wuerzburg with written informed consent of each patient. Participants in GSE56815 and GSE7429 are postmenopausal females and in GSE35957 are primary osteoporosis patients. A comprehensive list of CRGs was compiled based on existing literature (Huang et al., 2022; Li et al., 2022; Liu, 2022; Zhang et al., 2022). Finally, 31 CRGs were included in our research, containing AOC3, ATOX1, ATP7A, ATP7B, CCS, CD274, CDKN2A, COA6, COX11, COX17, CP, DBH, DBT, DLAT, DLD, DLST, FDX1, GCSH, GLS, H2C1, LIAS, LIPT1, LIPT2, LOXL2, MAP2K1, MAP2K2, MTCO2P12, MTF1, NFE2L2, NLRP3, PDE3B, PDHA1, PDHB, PDK1, SCO1, SLC25A3, SLC31A1, SLC31A2, SOD1, TYR, UBE2D1, UBE2D2, UBE2D3, UBE2D4, ULK1, ULK2, VEGFA.

The software R (https://www.bioconductor.org/) was used for data analysis. Datasets were filtered, background corrected, and normalized. The normalized dataset was subjected to CRGs with significant expression changes between osteoporosis patients and healthy controls. Differentially expressed CRGs were identified using the “limma” package with a threshold of |log_2_FC| > 0.5 and adjusted *P* < 0.05 (Benjamini–Hochberg FDR correction). Then, the significant correlations between every two CRGs were identified by “circlize” package.

### Immune infiltration analysis

Given that immune-cell infiltration such as B cells, NK cells, T cells, and macrophages was detected in osteoporosis patients (Gao et al., 2024; Keum et al., 2024), we also used the CIBERSORT method to estimate the percentage of different immune cell types that were present in the samples. Immune infiltration analysis bridges the gap between copper metabolism and bone remodeling by elucidating how CRGs modulate immune cell behavior (Dong et al., 2021). The CIBERSORT deconvolution algorithm, a machine learning methodology, relies on linear support vector regression, a computational method that ascertains the percentage of immune cells present in tissues or cells (Chen et al., 2018). It calculates the number of immune cells in a sample using RNA-sequence data (Newman et al., 2019). The link between CRG expression and immune cell infiltration in osteoporotic samples was investigated with the aid of this study. The work replicated the transcription characteristic matrix of 22 different kinds of immune cells using R and the CIBERSORT deconvolution technique (different situations of B cells, plasma cells, T cells, NK cells, monocytes, macrophages, dendritic cells, mast cells, eosinophils, and neutrophils). We compared the immune cell infiltration samples from the control group with the osteoporosis group. Meanwhile, the relationship between the differentially expressed CRGs and immune cells was explored. The “CIBERSORT” R script was used for data analysis in the software R. *P* < 0.05 was set as the significant criteria.

### Subclusters analysis of immune infiltration and GSVA analysis

The “ConsensusClusterPlus” tool in R software was utilized to categorize patients based on variations in CRG quality. Determining the inflection point of the sum of mistakes allowed for the selection and determination of the k value. The “limma” package was used to search the differential genes of the cluster patterns. A principal components analysis (PCA) map of the subclusters allowed us to see the geometric distance between them.

Gene Set Variation Analysis (GSVA) was conducted to clearly state the functional distinctions between the Cuproptosis subclusters. The “c2.cp.kegg.symbols.gmt” and “c5.go.symbols.gmt” files were downloaded for GSVA analysis from the online Molecular Signature Database (https://www.gsea-msigdb.org/gsea/msigdb/human/collections.jsp). We used the “limma,” “GSEABase,” and “GSVA” packages in R, with *P* < 0.05 considered significantly enriched. After that, a barplot was utilized to show how the two subclusters of genes involved in cuproptosis were distinct from one another in terms of the activity of their respective pathways.

### Genes screening based on WGCNA

By comparing these gene modules according to molecular subtypes and clinical characteristics, we were able to discover gene modules using Weighted Gene Co-expression Network Analysis (WGCNA). These genes were thought to be important contributors to cuproptosis’s participation in osteoporosis. We created a co-expression network of genes from normal people and osteoporosis samples using the “WGCNA” R program. Gene expression data is first normalized as part of the WGCNA approach, and then outliers are found using hierarchical clustering. A scale-free network architecture is obtained by choosing a soft-thresholding power β. Next, an adjacency matrix is calculated and transformed into a topological overlap matrix, which forms the foundation for dynamic tree cutting-based module discovery. The next step is to calculate the eigengenes of each module, which are then connected with clinical features to find the modules that are linked to the desired attributes. Lastly, network visualization helps identify genes inside important modules.

The significant module filter was set to 0.8 and the significant gene filter to 0.5 for this investigation. Two groups of osteoporosis individuals were identified based on differentially expressed CRGs, and genes based on WGCNA were studied from the perspectives of both osteoporosis and normal patients. The genes we utilized for additional analysis were found at the intersection of two findings.

### Machine learning approaches to screen final core genes

To develop prediction models for osteoporosis, we used machine learning methods such as Random Forest (RF), Support Vector Machine (SVM), XGBoost, and Generalized Linear Model (GLM) to further pick the final possible core genes connected to cuproptosis. RF is an ensemble prediction method that evaluates each variable’s importance and can process large amounts of input data. The repeatedcv method was used and number of trees is five in this study. This method creates a large number of decision trees during training, and the class mode (Yang et al., 2024). During the SVM model’s training phase, a svmRadial kernel approach was applied to address the non-linear connection between variables. Setting a threshold between two classes so that label prediction based on one or more feature vectors is possible is the aim of the potent SVM technique (Absar et al., 2022). XGBoost is an open-source program that is frequently used to develop machine learning algorithms under the Gradient Boosting framework in regression and classification applications. In data science challenges, it is renowned for its performance, accuracy, and speed (Nguyen et al., 2024). We used the xgbDART method to complete the analysis. With the ability to include response variables with error distribution models other than normal distributions, GLM models expand on the capabilities of classic linear regression. By using a link function to connect these variables to linear predictors, it can be used for a variety of data types and is a key instrument in statistical data analysis (Mao et al., 2024). In GLM model, the glm method and binomial family were adopted. Finally, we selected the most precise method based on the sensitivity, specificity, square of residuals, and root mean square error. These genes may serve as potential biomarkers or therapeutic targets. R packages “caret,” “DALEX,” “ggplot2,” “randomForest,” “kernlab,” “xgboost,” and “pROC” finished the four machine learning techniques.

### Construction and validation of osteoporosis predictive model based on core genes

We developed a nomogram evaluation method based on core genes to forecast the likelihood of osteoporosis and identify the treatment approach. The agreement between the observed and expected values was assessed using calibration curves. To assess our model’s therapeutic advantages, we also ran decision curve studies. Decision curve analysis is a technique used to calculate the net benefit of employing a predictive model for decision-making to assess the therapeutic value of the models. With the use of receiver operating characteristic (ROC) curves, the effectiveness of the machine learning models was assessed. To evaluate the prediction accuracy of the models in differentiating osteoporotic samples from controls, the area under the curve (AUC) was determined. All the analysis was conducted by the “pROC,” “rms,” and “rmda” R packages.

Finally, two different external datasets (GSE7429 and GSE35957) were used to confirm the core genes’ expression levels and significance. The ROC was used to evaluate the performance of core genes in predicting the groups.

In addition, mouse bone mesenchymal stem cells (BMSCs) experiment was conducted to testify the different expressions of core genes. The protocol was approved by the Ethics Committee of Shanghai Second People’s Hospital (No.: 2023-N015; date: 18 September 2023). Twenty C57BL/6J mice [bought from Lingchang BioTech Co., Ltd (Shanghai, China)]. were fed a standard diet in a facility with a temperature range of 22°C–25°C, 50% relative humidity, and 12-h light/dark cycles for 1 week prior to the experiment. Ten mice were divided into the control group and received a sham surgery. The other ten mice as the osteoporosis model group received the ovariectomy. BMSCs were derived from the femur and tibia after 12 weeks and then cultured in αMEM supplemented with 10% fetal bovine serum. The total RNAs were extracted to complete the Quantitative Real-time PCR (qPCR). Table 1 contains the specific primers for each gene. Sangon Biotech Co., Ltd. created the PCR primers using the online tool Primer BLAST from the National Center for Biotechnology Information (Shanghai, CN). Following the manufacturer’s instructions, reverse transcription was carried out using PrimeScript RT Reagent Kit (Takara Bio, Shiga, JP). TB Green Premix EX Taq with fluorescence quantitative PCR instrument Applied Biosystems (Thermo Fisher Scientific, Waltham, USA) was used to test the relative RNA expression.

**TABLE 1:** List of primers for real-time PCR analysis.

Gene Symbol	Accession Number	Oligonucleotide Sequence	
GPR27	NM_008158.2	Forward	5’ATGGCGAACGCTAGTGAGC3’
		Reverse	5’GGCGGTCGTGGATGAAGAAG3’
PDE8A	NM_008803.3	Forward	5’CCGAGCATCCACACTTCCG3’
		Reverse	5’TCAGCTACTGATACCTTCGAGG3’
NIF3L1	NM_022988.3	Forward	5’AAGAAATGCTGGGTGTTCACTT3’
		Reverse	5’GAGGGTCCCTGTCTGTCTCA3’
CIR1	NM_025854.4	Forward	5’GGGAAGTCATTCGCCAATTTCA3’
		Reverse	5’CACGTTCATCTCCCATAAGCAAT3’
VPS35	NM_022997.5	Forward	5’GCTGTGAAGGTTCAGTCATTCC3’
		Reverse	5’GTCAGGTAGACCTCCAAGTAGT’

### Enrichment analysis and potential drugs

In order to analyze the biological functions and pathways involved in core genes related to cuproptosis, enrichment analysis was performed on Enrichr (https://maayanlab.cloud/Enrichr/#, assessed on 25 April 2024) (Evangelista et al., 2023).Gene functions and interactions, such as biological processes (BP), molecular functions (MF), and cellular components (CC), are frequently described using gene ontology (GO) enrichment analysis (Pomaznoy et al., 2018). A common method for storing data regarding genomes, biological pathways, and illnesses is the Kyoto Encyclopedia of Genes and Genomes (KEGG) pathway enrichment analysis (Kanehisa et al., 2017). An essential Elixir resource, the Reactome Knowledgebase (https://reactome.org), offers carefully selected molecular information on a wide range of physiological and pathological biological processes in humans, including both inherited and acquired disease processes (Gillespie et al., 2022). WikiPathways is a collaborative biological pathway database (https://www.wikipathways.org) that promotes open research practices and removes obstacles to material access and usage. It is now being used by more initiatives, projects, tools, and content creators. This is particularly true for groups of people that focus on certain biological processes, such as lipid metabolism and rare diseases (Martens et al., 2021). In addition, we also analyze the enrichment of core genes in GWAS database (https://gwas.mrcieu.ac.uk/, P-value <0.05, linkage disequilibrium with an *r* ^2^ threshold of 0.8 and a ±500 kb window,), human phenotype (https://hpo.jax.org/app/), Jensen tissue (https://tissues.jensenlab.org/Search), human cell (https://hubmapconsortium.org/), and potential drug targets (https://druggablegenome.net/). The protein–protein interaction (PPI) network was also analyzed (https://string-db.org/).

### Statistical analysis

All statistical tests were completed in the R software version 4.3.2. The correlation between the variables was determined using Pearson’s or Spearman’s correlation test. qPCR data were analyzed using unpaired two-tailed Student’s t-tests (GraphPad Prism v10.0). *P*-value less than 0.05 was set to be statistically significant.

## Results

### The landscape of genetic variation of CRGs in osteoporosis

**FIGURE 1:**
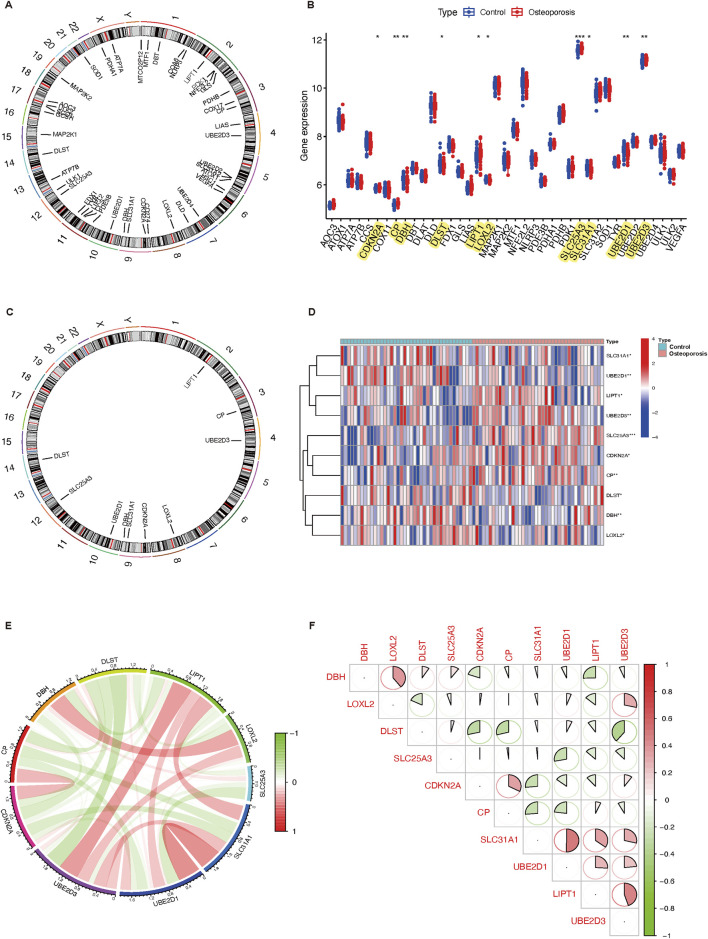
Expression characteristics and gene localization of CRGs. **(A)** The position of 31 CRGs on the chromosome. **(B)** Boxplot showing differences in the expression of CRGs in osteoporosis and control participants, with significant differences in the expression of 10 genes. **(C)** The chromosome distribution of number variation changes among 10 differential expressed CRGs. **(D)** Heatmap showing the expression characteristics of 10 CRGs in in osteoporosis and control participants. Red represents high expression levels while blue represents low expression levels. **(E,F)** Spearman correlation analysis of CRGs; positive correlation is represented by red, while negative correlation is represented by green. Bigger area means stronger correlation. *P < 0.05, **P < 0.01, and ***P < 0.001. CRGs, cuproptosis-related genes.

The GSE56815 dataset was adopted to determine the expression levels of 31 cuproptosis-related genes in osteoporosis and normal participants. The location of universal copy number variation changes for the 31 genes was presented in Figure 1A. The distribution of differentially expressed 10 CRGs between normal controls and osteoporosis patients was visualized by a boxplot and a heatmap in Figures 1B,D. It showed that in osteoporosis patients, CP (*P* < 0.01), LIPT1 (*P* < 0.05), SLC25A3 (*P* < 0.001), and UBE2D3 (*P* < 0.01) were upregulated, while the expression levels of CDKN2A (*P* < 0.05), DBH (*P* < 0.01), DLST (*P* < 0.05), LOXL2 (*P* < 0.05), SLC31A1 (*P* < 0.05), and UBE2D1 (*P* < 0.01) were downregulated. The chromosome location demonstrated that LIPT1 on chromosome 2; CP on chromosome 3; UBE2D3 on chromosome 4; LOXL2 on chromosome 8; CDKN2A, DBH, and SLC31A1 on chromosome 9; UBE2D1 on chromosome 10, SLC25A3 on chromosome 12; DLST on chromosome 14 (Figure 1C). Moreover, a CRG interaction network was constructed to illustrate their interconnections (Figures 1E,F). There was a substantial and strong positive correlation found between the expression of SLC31A1 and UBE2D1, a significant positive correlation between LIPT1 and UBE2D3A, and a positive correlation between DBH and LOXL2 expression. However, a significant negative correlation was found between DLST and UBE2D3.

As the research above indicates, osteoporosis displays a diverse landscape of CRG genetic and expression alterations. This demonstrated that CRGs play an essential role in osteoporosis formation and progression.

### Immune infiltration analysis

**FIGURE 2:**
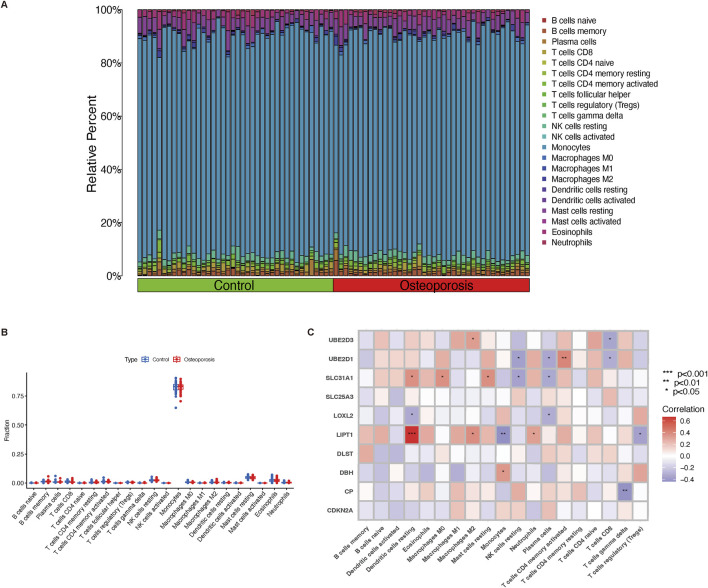
Immune cell infiltration and correlation analysis. **(A)** The relative percentage of 22 immune cell subtypes between osteoporosis and control participants. **(B)** Boxplot illustrates different fractions of 22 immune cells in osteoporosis and control participants. **(C)** The correlation analysis of immune cells and differential expressed CRGs. Red and blue represent positive and negative correlation, respectively. *P < 0.05, **P < 0.01, and ***P < 0.001. CRGs, cuproptosis-related genes.

CIBERSORT algorithm and GSVA method revealed a differential expression matrix of immune cell subtypes in osteoporosis patients compared to controls. The distribution of 22 immune cells in each sample is represented in the heatmap (Figure 2A). It showed that the relative percent in the monocytes is the highest. There is no significant difference in immune cells between osteoporosis and control participants (Figure 2B). Among the differentially expressed CRGs, immune infiltration was significantly correlated to seven genes (UBE2D3, UBE2D1, SLC31A1, LOXL2, LIPT1, DBH, and CP). Figure 2C represented the detailed correlation analysis of UBE2D3 with positive correlation of macrophages M2 (*P* < 0.05), negative correlation of CD8^+^ T cells (*P* < 0.05); UBE2D1 with positive correlation of CD4^+^ T cells memory activated (*P* < 0.01), negative correlation of resting NK cells, plasma cells, and CD8^+^ T cells (*P* < 0.05); SLC31A1 with positive correlation of resting dendritic cells, macrophages M0, and resting mast cells (*P* < 0.05), negative correlation of resting NK cells and plasma cells (*P* < 0.05); LOXL2 with negative correlation of resting dendritic cells and plasma cells (*P* < 0.05); LIPT1 with positive correlation of resting dendritic cells (*P* < 0.001), macrophages M2 (*P* < 0.05), and neutrophils (*P* < 0.05), negative correlation of monocytes (*P* < 0.01); DBH with positive correlation of monocytes (*P* < 0.05); CP with negative correlation of gamma delta T cells (*P* < 0.01).

### Subclusters analysis of immune infiltration

**FIGURE 3:**
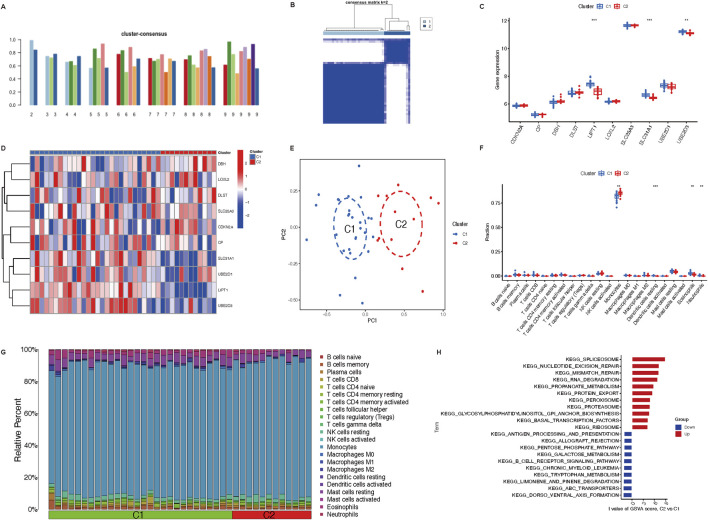
Immune cells infiltration of the two distinct clusters and GSVA analysis. **(A)** Various consensus score of different k value (from two to 9). **(B)** The consensus matrix heatmap (k = 2) exhibits the two clusters’ correlation region. **(C,D)** Boxplot and heatmap depict the expression of 10 CRGs in GeneClusters. **(E)** Remarkable transcriptome differences between the two clusters based on PCA. **(F)** Boxplot illustrates different fractions of 22 immune cells in two clusters. **(G)** Heatmap shows the relative percentage of 22 immune cell subtypes between two clusters. **(H)** GSVA analysis represents regulated pathways in two clusters. **P < 0.01, and ***P < 0.001. CRGs, cuproptosis-related genes; PCA, principal components analysis; GSVA, Gene Set Variation Analysis.

Consistent clustering was adopted to better clarify the clinical significance or biological pattern of CRGs. According to the expression levels of ten CRGs, osteoporosis samples were divided into subgroups. As discovered, the best stability of clustering can be offered with k = 2 (Figure 3A). Only two colors were shown with k = 2 in the tracking plot (Supplementary File 1). In addition, the relative change region below the cumulative distribution function curve and cumulative distribution function diagram were shown in Supplementary Files 2,3. Osteoporosis samples were divided into cluster 1 (n = 28) and cluster 2 (n = 12) of the two different clusters (Figure 3B) (Supplementary File 4). Following the boxplot (Figure 3C) and heatmap (Figure 3D), the cluster one group exhibited increased expression levels of UBE2D3 (*P* < 0.001), SLC31A1 (*P* < 0.001), and LIPT1 (*P* < 0.01). Moreover, in accordance with CRG expression levels, PCA indicated that the two groups showed a remarkable difference in CRG transcriptional profiles (Figure 3E). According to immune infiltration analysis for osteoporosis patients, the cluster two group showed low innate immune cell infiltration, including cells such as resting dendritic cells (*P* < 0.001), eosinophils (*P* < 0.01), and neutrophils (*P* < 0.01); high innate immune cell infiltration, only monocytes (*P* < 0.01) (Figure 3F). The heatmap (Figure 3G) depicts the distribution of 22 immune cells in each sample, with the macrophage having the highest relative percent.

The GSVA analysis discovered that pathways (spliceosome, nucleotide excision repair, mismatch repair, RNA degradation, propanoate metabolism, protein export, peroxisome, proteasome, glycosylphosphatidylinositol GPI anchor biosynthesis, basal transcription factors, ribosome) were upregulated in cluster 2; however, pathways (antigen processing and presentation, allograft rejection, pentose phosphate pathway, galactose metabolism, B cell receptor signaling pathway, chronic myeloid leukemia, tryptophan metabolism, limonene and pinene degradation, ABC transporters, dorso ventral axis formation) were downregulated in cluster 1 (Figure 3H).

### Genes screening based on WGCNA

**FIGURE 4:**
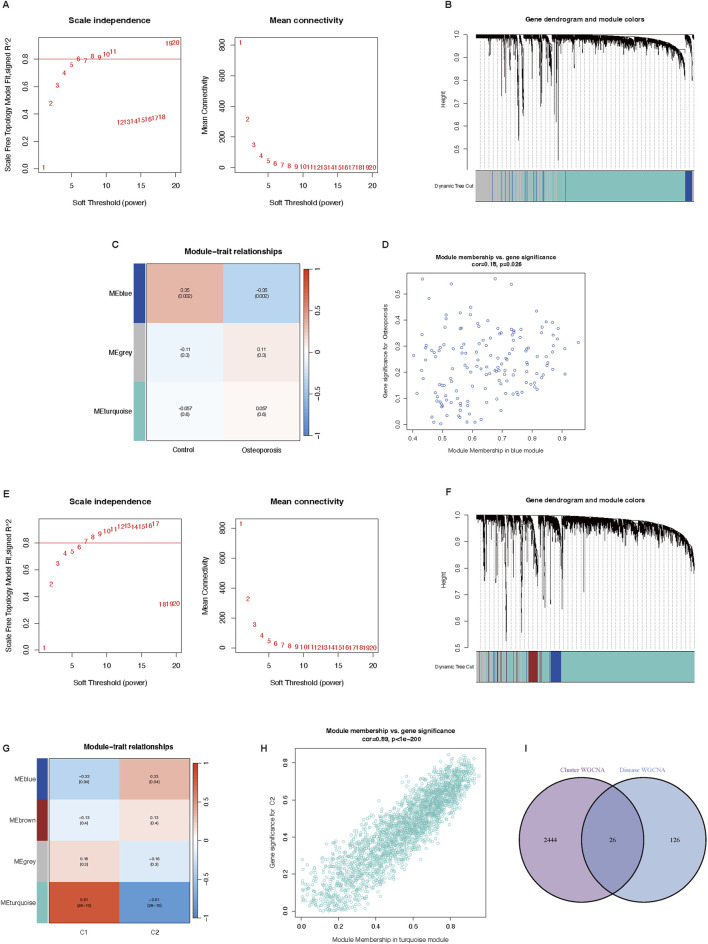
Potential key genes screening based on WGCNA. **(A–D)** Co-expression network of differential expressed genes between osteoporosis patients and normal individuals. **(A)** Soft threshold analysis in osteoporosis. **(B)** Module correlations in osteoporosis. **(C)** Module-trait correlation heatmap, red is positive correlation, blue is negative correlation. **(D)** Scatter plot of blue module. **(E–H)** Co-expression network of differential expressed genes between two cuproptosis related osteoporosis clusters. **(E)** Soft threshold analysis in osteoporosis. **(F)** Module correlations in osteoporosis. **(G)** Module-trait correlation heatmap, red is positive correlation, blue is negative correlation. **(H)** Scatter plot of blue module. **(I)** Venn plot of genes in cluster WGCNA and osteoporosis WGCNA. WGCNA, Weighted genes correlation network analysis.

The WGCNA analysis of genes between osteoporosis and normal participants was performed first. As shown in Figure 4A, the analysis of soft threshold selection revealed that gene associations were maximally consistent with the scale-free distribution when β = 11 (scale free R^2^ = 0.8). Based on selected soft-thresholding power, a hierarchical clustering tree was established to cluster high co-expression genes into the same module and color code them (Figure 4B). Then, a total of three modules were identified in the weighted gene co-expression network by setting the minimum number of genes in a module to 20. We found that MEblue module (*P* = 0.002) was positively correlated with osteoporosis in non-gray modules by Spearman correlation analysis (Figure 4C). Genes in the MEblue module were further used in the analysis, including 152 genes (Figure 4D). It showed a significant correlation between genes of osteoporosis and blue module membership (cor = 0.18, *P* = 0.026). We also selected 1000 genes randomly to draw a network heatmap to show the correlation among genes in the same module (Supplementary File). More golden yellow meant stronger related. It showed a relatively high correlation in the MEturquoise module. The detailed information about the relationship between gene significance and module membership was summarized in Supplementary File 6.

Then we conducted the WGCNA analysis of genes in osteoporosis patients between cluster one and cluster two based on differentially expressed CRGs. The results of the soft threshold selection analysis, as presented in Figure 4E, indicated that gene correlations were maximally compatible with the scale-free distribution when β = 9 (scale free R^2^ = 0.8). A hierarchical clustering tree was created to color code and group high co-expression genes into the same module based on the chosen soft-thresholding power (Figure 4F). Through Spearman correlation analysis, we discovered that C2 had the highest connection with the MEturquoise module (*P* < 0.001) (Figure 4G). 2,470 genes of the MEturquoise module were further analyzed (Figure 4H). It showed a significant correlation between cluster two genes and turquoise module membership (cor = 0.89, *P* < 0.001). Additionally, 1000 randomly chosen genes were used to create a network heatmap that displays the association between the genes in the same module (Supplementary File 7). In the MEturquoise module, the correlation was comparatively high. The Supplementary File 8 provided a full summary of the facts about the link between the gene importance of two clusters and module membership.

Finally, we obtained 26 potential key genes (Supplementary File 9) by intersecting 152 genes of disease WGCNA and 2,470 genes of cluster WGCNA in a Venn plot (Figure 4I).

### Core genes related to cuproptosis through machine learning approaches

**FIGURE 5:**
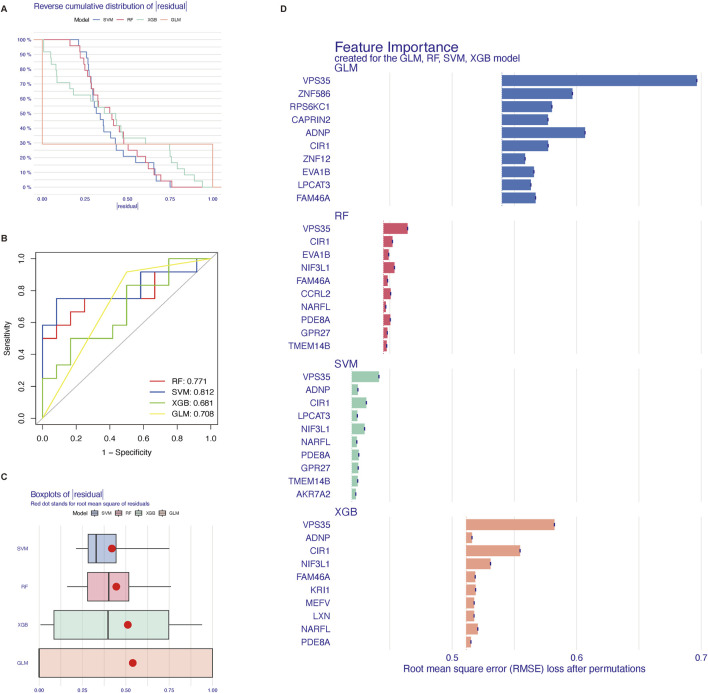
Four machine learning methods to screen core genes related to cuproptosis. **(A)** reverse cumulative distribution of residuals. **(B)** ROC curves as a function of the values of observed sensitivity and specificity among four methods. **(C)** Boxplot of the residual distribution. **(D)** The importance of the four models and 10 top genes each. SVM, support vector machine; RF, random forest; XGB, XGBoost; GLM, generalized linear model.

Reverse cumulative distribution of residuals (Figure 5A), ROC curve analysis (Figure 5B), and Boxplots of residuals (Figure 5C) demonstrated that SVM displayed notably high predictive capability. In Figure 5A, the SVM model demonstrated the best performance among the four models. The majority of the samples had small absolute residuals, as indicated by the steeper curve in the low residual range. This steepness suggested that the SVM model achieved the highest accuracy. Therefore, the SVM model was highly accurate and reliable for this dataset. The RF model showed good performance with a relatively concentrated residual distribution. Although the curve was not as steep as that of the SVM model, the RF model still performed well overall. Its predictive accuracy was high, making it a strong contender, albeit slightly less optimal than the SVM model for this particular dataset. The XGB model exhibited moderate performance, with a relatively flat curve, especially in the low absolute residual range. This flatness indicated that the XGB model had larger prediction errors for many samples. Despite its general robustness, the XGB model’s performance on this dataset was average, suggesting that it might require further parameter tuning or feature engineering to improve its accuracy. The GLM model had the poorest performance, as shown by the flattest curve among the four models. The wide distribution of residuals across the entire range suggested that the GLM model had relatively high prediction errors and lower accuracy. Consequently, the GLM model was not suitable for this predictive task on the given dataset. In Figure 5B, the AUC value was a single scalar value that summarized the overall performance of the model. A higher AUC indicated better performance. The SVM model, with the highest AUC of 0.812, was the best performer for distinguishing between the classes in this dataset. In Figure 5C, the boxplot analysis showed that SVM and RF had relatively low variability in residuals and potentially lower root mean square of residuals (RMSR), making them strong candidates for accurate predictions. XGB showed moderate variability and RMSR, suggesting it had decent performance but with more variability. GLM had the highest variability in residuals and the highest RMSR, indicating it was the least reliable model among the four in this context. According to the root mean square error loss after permutations, the results of the SVM method were selected (Figure 5D). In total, we showed ten core genes related to cuproptosis (VPS35, ADNP, CIR1, LPCAT3, NIF3L1, NARFL, PDE8A, GPR27, TMEM14B, and AKR7A2) by the SVM method.

### Construction and validation of osteoporosis model based on core genes related to cuproptosis

**FIGURE 6:**
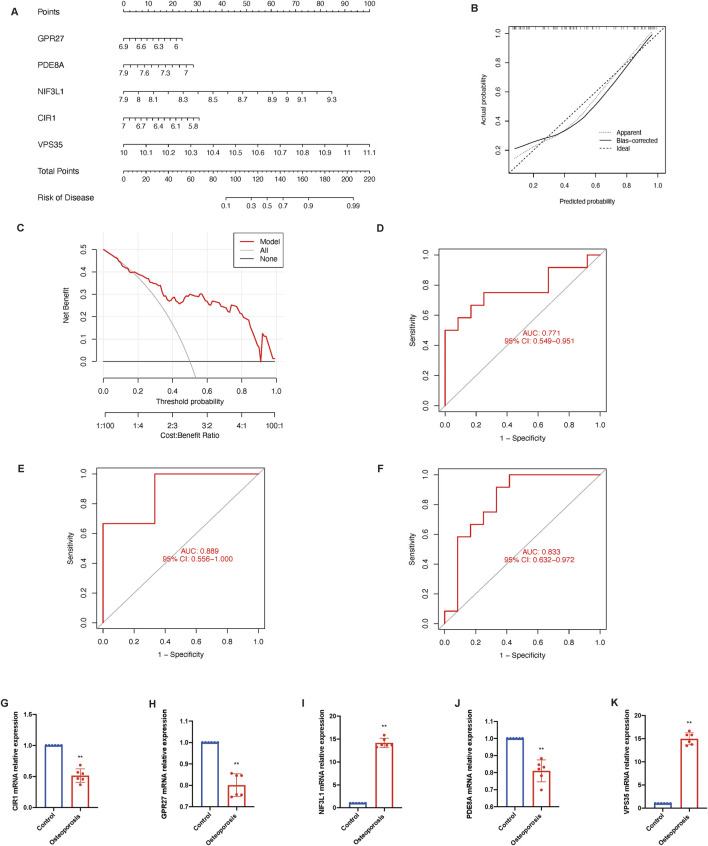
Construction and validation of osteoporosis model. **(A)** Nomogram of the predictive model based on top five core genes related to cuproptosis. **(B)** Calibration curves shows that the nomogram model may be an ideal predictive model for osteoporosis. **(C)** Decision-curve analysis is used to determine the clinical utility of the risk prediction nomograms. **(D)** ROC curve indicates the diagnostic performance of the model. ROC curve shows the diagnostic performance by this model in an external dataset GSE7429 **(E)** and GSE35957 **(F)**. **(G–K)** The mRNA relative expression of CIR1, GPR27, NIF3L1, PDE8A, and VPS35. Data presented as mean ± standard deviation; statistical significance determined by unpaired t-test (**P < 0.01). AUC, area under the curve; ROC, receiver operating characteristic.

Genes were ranked by SVM-derived importance scores, and the top five (GPR27, PDE8A, NIF3L1, CIR1, and VPS35) were selected due to their cumulative contribution (85.4%) to model accuracy. To predict the probability and treatment targets of osteoporosis, we constructed a nomogram evaluation mode based on these top five core genes (Figure 6A). The calibration curves (Figure 6B) and decision-curve analysis (Figure 6C) proved the nomogram model to be an ideal predictive model for osteoporosis. We further evaluated the diagnostic values of these biomarkers. The AUC value of the ROC curve was 0.771 for this model (Figure 6D). Then we performed an external validation by GSE7429 and GSE35957, and the AUC value of the ROC curve was 0.889 and 0.833 respectively (Figures 6E,F), suggesting biological relevance of the identified cuproptosis-related genes despite demographic variations. Additionally, the mRNA relative expression of CIR1 (Figure 6G), GPR27 (Figure 6H), and PDE8A (Figure 6J) were reduced in the osteoporosis group, while NIF3L1 (Figure 6I) and VPS35 (Figure 6K) were increased.

### Enrichment of top five core genes related to cuproptosis and potential drugs

**FIGURE 7:**
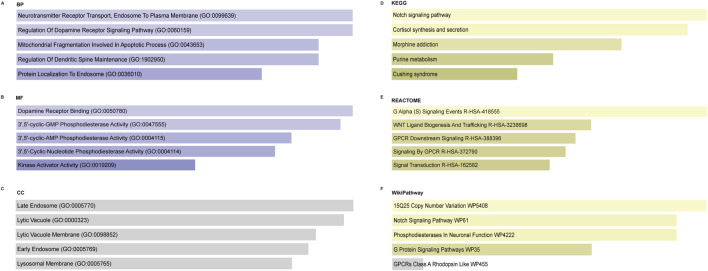
Enrichment analysis of the top five core genes related to cuproptosis. **(A)** Significantly enriched biological processes. **(B)** Significantly enriched molecular functions. **(C)** Enrichment of cellular components. **(D)** Significantly enriched KEGG pathway. **(E)** Significantly enriched REACTOME pathway. **(F)** Significantly enriched WikiPathway. KEGG, Kyoto Encyclopedia of Genes and Genomes; BP, biological process; MF, molecular function; CC, cellular component.

GO enrichment analysis was performed on the top five core genes related to cuproptosis (GPR27, PDE8A, NIF3L1, CIR1, and VPS35). The BP enriched by these differential genes was mitochondrial fragmentation involved in the apoptotic process, neurotransmitter receptor transport, endosome to plasma membrane, and regulation of the dopamine receptor signaling pathway (Figure 7A). MF analysis indicated that these genes were enriched in dopamine receptor binding, phosphodiesterase activity, and kinase activator activity (Figure 7B). No significance was found in the CC enrichment (late endosome, lytic vacuole, lytic vacuole membrane, early endosome, and lysosomal membrane) (Figure 7C). KEGG pathway enrichment analysis revealed that these differential genes significantly enriched the top five pathways: the Notch signaling pathway, cortisol synthesis and secretion, morphine addiction, purine metabolism, and Cushing syndrome (Figure 7D). In addition, REACTOME (Figure 7E) and WikiPathway (Figure 7F) analyses were also performed. The results revealed that the core genes were significantly enriched in WNT ligand biogenesis and trafficking, signal transduction, the Notch signaling pathway, and so on.

**FIGURE 8:**
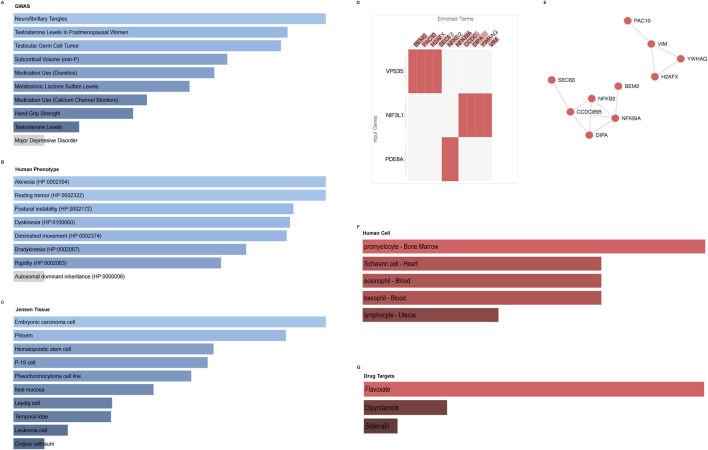
Enrichment of the top five core genes in different databases. **(A)** Enrichment of GWAS database. **(B)** Human tissue enrichment. **(C)** Jensen cell enrichment. **(D,E)** PPI network. **(F)** Human cell enrichment. **(G)** Potential drug targets.

We also enriched the five genes in the GWAS database (neurofibrillary tangles, hand grip strength, and so on) (Figure 8A), the human phenotype database (akinesia, dyskinesia, diminished movement, bradykinesia, and so on) (Figure 8B), and the Jensen tissue database (embryonic carcinoma cell, phloem, and so on) (Figure 8C). As shown in Figure 8D, the PPI network indicated that VPS35 has a correlation with four proteins (BEM2, PAC10, H2AFX, and SEC63), NIF3L1 with four proteins (CCDC85B, DIPA, YWHAQ, and VIM), and PDE8A with two proteins (NFKB2 and NFKBIA). Then, the relationship of the ten proteins was conducted and represented in Figure 8E. It suggested that NFKB1A and CCDC85B had the most connection with other proteins. In addition, the five core genes were mainly enriched in bone marrow cells (Figure 8F). According to the genes, three drugs (flavoxate, dipryridamole, and sildenafil) might play an important role in treating osteoporosis (Figure 8G).

## Discussion

Our investigation utilizing the GSE56815 dataset significant genetic and expression dysregulation of CRGs in osteoporosis, highlighting the complexity of this disease. Key findings include the downregulation of CDKN2A, DBH, DLST, LOXL2, SLC31A1, and UBE2D1, alongside the upregulation of CP, LIPT1, SLC25A3, and UBE2D3. These results are in line with other researchers’ hypotheses on a possible connection between osteoporosis and CDKN2A (Curtis et al., 2017), DBH (Ji et al., 2023), LOXL2 (Han et al., 2018), CP (Karakas et al., 2016), SLC25A3 (Kang et al., 2021), and UBE2D3 (Shao et al., 2020). Cellular senescence and aging have been connected to CDKN2A, which is well-known for its function in controlling the cell cycle (Jiao et al., 2018). Its downregulation in individuals with osteoporosis may indicate a reduction in cellular senescence, which would boost osteoclast activity and bone resorption, weakening bone structure. Norepinephrine, which has been demonstrated to affect bone remodeling, is synthesized by DBH (Liu et al., 2024). Decreased norepinephrine levels resulting from decreased DBH expression may have adverse effects on osteoblast activity and bone formation. The integrity of the bone matrix is maintained by the cross-linking of collagen fibers, which is facilitated by LOXL2 (Bates et al., 2023). Reduced LOXL2 levels may lead to a weakened bone matrix, increasing the risk of bone fractures. Ceruloplasmin, or CP, possesses antioxidant qualities and is involved in iron metabolism (Warjukar et al., 2024). A compensatory mechanism to offset increased oxidative stress in osteoporotic bones—which might result in bone degradation—might be indicated by overexpression of CP. Because SLC25A3 is essential to mitochondrial function, its overexpression in osteoporotic bones may be a reaction of the cells to elevated energy requirements or malfunction in the mitochondria (Kucukcongar Yavas et al., 2024). The control of immunological response and protein breakdown is suggested by UBE2D3’s participation in ubiquitination (Yalcin et al., 2023). Its upregulation may be associated with the immune system’s effort to control inflammation in bones that have osteoporosis. DLST SLC31A1, LIPT1, and UBE2D1 are reported to regulate cuproptosis in sepsis (Wang et al., 2024), hepatocellular carcinoma (He et al., 2024), multiple myeloma (Zhu Y. et al., 2023), thyroid carcinoma (Yu et al., 2024), oral squamous cell carcinoma (Yuan et al., 2023), rheumatoid arthritis (Jiang et al., 2023), periodontitis (Liu S. et al., 2023), gastric cancer (Zhu X. et al., 2023), colorectal cancer (Yang W. et al., 2023), and myocardial infarction (Yang et al., 2024). Immunological system involvement is a commonality across all these disorders. *In vivo* and *in vitro* experiments should be done to verify the accurate roles of these genes in the pathology of osteoporosis.

Emerging research increasingly highlights the critical role of cuproptosis in immune regulation (Yan et al., 2022). The hypothesis that cuproptosis and the immune system are associated with the pathophysiology of osteoporosis is strengthened by our data, which supports these findings by demonstrating a substantial correlation between CRGs and monocyte infiltration in osteoporosis. Monocytes and macrophages are essential for bone remodeling, and their dysregulation can contribute to osteoporosis (Xu et al., 2024). Notably, UBE2D3 expression exhibits a positive correlation with M2 macrophages and a negative correlation with CD8^+^ T cells. This pattern reflects a complex interplay between bone degradation and immune response, potentially indicative of a chronic inflammatory state within the osteoporotic bone microenvironment.

Additionally, we employed unsupervised cluster analysis to show the various cuproptosis regulation patterns in osteoporosis patients based on the expression landscapes of CRGs. Two different clusters connected to cuproptosis were found. In the cluster two osteoporosis group, we observed that the majority of CRGs were downregulated. Furthermore, only monocytes exhibited a significant level of innate immune cell infiltration in the cluster two group. Growing evidence also highlights the critical role that monocytes play in cuproptosis (Liu Z. et al., 2022; Xu et al., 2022). In addition, DNA damage drives accelerated bone aging via an NF-κB-dependent mechanism; however, nucleotide excision and mismatch repair can improve this situation (Chen et al., 2013). B cell contributes to the inflammation to destroy the equilibrium of bone metabolism (Frase et al., 2023). One possible target for osteoporosis therapy is bone immunity, which controls osteoclast development and bone resorption (Wang X. et al., 2022). Collectively, these findings suggest that Cluster 2 may be associated with poorer clinical outcomes, warranting further investigation. This variation emphasizes the necessity of personalized medicine approaches for osteoporosis treatment. The interplay between CRGs and immune cells, particularly monocytes and macrophages, highlights novel opportunities for immunomodulatory therapies.

Four machine learning techniques are utilized to further evaluate the 26 cuproptosis-related genes identified through WGCNA. Next, five hub genes (GPR27, PDE8A, NIF3L1, CIR1, and VPS35) with the highest prognostic significance were selected to construct a predictive nomogram. This developed model was verified in two external datasets, GSE7429 (AUC = 0.889) and GSE35957 (AUC = 0.833), indicating its potential clinical utility in osteoporosis risk stratification. Mechanistically, cell proliferation can be impacted by GPR27 via the MAPK/ERK pathway, which is critical for bone growth and maintenance (Wang H. et al., 2022). PDE8A functions as a novel protective target in central nervous system inflammation, where its expression in pathogenic Teff cells suggests potential roles in bone inflammatory responses and remodeling processes (Epstein et al., 2021). VPS35 regulates receptor activator of NF-κB trafficking, signaling, and function to preserve healthy bone mass and structure (Xia et al., 2013). However, studies about NIF3L1 and CIR1 are rare; we just know that NIF3L1 appears in both osteoporosis and osteoarthritis patients (Li et al., 2019), and CIR1 is a transcription factor that regulates iron acquisition and use (Xue et al., 2024). Further exploration could be done in this aspect.

Functional enrichment analysis of the top five core genes provides deeper insight into the molecular processes that cuproptosis may affect in osteoporosis. The discovered pathways, like the apoptotic process, the Notch signaling pathway, WNT ligand biogenesis and trafficking, offer fascinating linkages to bone metabolism and might inspire new lines of inquiry. These pathways also suggest a link to cuproptosis and osteoporosis (Chen et al., 2009; Xu et al., 2022; Yang L. et al., 2023; Zaidi et al., 2023). Apoptosis, also known as programmed cell death, is an essential mechanism that balances the production and resorption of bones to preserve bone homeostasis (Jilka et al., 2013). Osteoporosis and other bone disorders can result from dysregulation of apoptosis (Weinstein and Manolagas, 2000; Mollazadeh et al., 2015). According to studies, osteoblasts, or bone-forming cells, and osteocytes, or mature bone cells, undergo excessive apoptosis, which decreases bone production and increases bone fragility (Florencio-Silva et al., 2015). Because core genes are enriched in the apoptotic process, changes to these genes may upset the equilibrium between bone creation and resorption, resulting in osteoporosis. Through controlling osteoblast and osteoclast (bone-resorbing cell) differentiation and activity, the Notch signaling system plays a crucial role in bone growth and remodeling (Jakovljevic et al., 2023). Increased Notch signaling has been shown to suppress osteoblast development, which lowers bone production and increases bone resorption (Yoshida et al., 2022). Given that core genes are involved in the Notch signaling system, cuproptosis may affect osteoporosis by modulating this route and perhaps interfering with normal processes involved in bone remodeling. The production and regeneration of new bone depend on the WNT signaling system. It increases the survival and functionality of mature osteoblasts and encourages mesenchymal stem cells to differentiate into osteoblasts (Marini et al., 2023). Osteoporosis can result from mutations or dysregulation in WNT signaling components, which can reduce bone production and increase bone resorption (Gao et al., 2023). Cuproptosis-related genes VPS35 impairs the availability and activity of WNT ligands, which might alter bone metabolism and contribute to osteoporosis (Chen et al., 2016; Chiu et al., 2020).

In addition, these genes also relate to clinical symptoms like akinesia, dyskinesia, diminished movement, and bradykinesia, which are commonly observed in neurodegenerative diseases like Parkinson’s disease (Hayes, 2019). This neuro-bone axis suggests that cuproptosis-related pathways may have systemic effects on neuromuscular coordination, emphasizing their complex physiological roles (Cao et al., 2023). The similarity in symptoms between neurological and bone disorders emphasizes how complex cuproptosis is and how many physiological systems it may affect. Furthermore, prospective drugs that may target these key genes include sildenafil, dipyridamole, and flavoxate, as revealed by the enrichment analysis. These medications’ established pharmacological actions may make them useful for treating osteoporosis. For instance, it has been demonstrated in animal models that the phosphodiesterase inhibitor sildenafil enhances bone repair and promotes bone production (Bereket et al., 2018). The vasodilatory and anti-inflammatory properties of dipyridamole and flavoxate may synergistically improve bone microcirculation and reduce inflammatory bone loss (Baert, 1974; Macatangay et al., 2020). Prospective drugs may target CRGs through dual osteoimmunomodulatory mechanisms, particularly in patients with dysregulated copper metabolism or immune-driven bone loss. The discovery of possible medications using enrichment studies opens possibilities for pharmacological intervention, which might be essential for treating osteoporosis given the enrichment of these genes in bone marrow cells. However, their effects on bone metabolism and CRG expression require experimental validation in cellular or animal models before any translational implications can be drawn.

There are certain restrictions on this article. First, while FDR correction was applied, the exploratory nature of bioinformatics analyses warrants independent validation to minimize false positives. Secondly, our qPCR validation of core genes in mouse BMSCs provided preliminary transcriptional evidence but lacked protein-level and *in vivo* functional data. The translational relevance to human osteoporosis requires further investigation. Third, different cell types (PBMCs, B lymphocytes, and MSCs) across datasets introduces complexity into comparative analyses. Future validations in tissue-matched cohorts are essential. Finally, the validation cohorts (GSE7429 and GSE35957) had limited sample sizes and heterogeneous patient characteristics (e.g., menopausal status, osteoporosis etiology), which may confound the generalizability of our predictive model. Our bioinformatic model identifies hypothetical cuproptosis-related biomarkers and therapeutic targets. While machine learning and cross-dataset validation support their relevance, these predictions require empirical confirmation. Based on this, we have a detailed future research agenda as follows: to validate the predictive model in diverse clinical settings; to evaluate the model’s feasibility and effectiveness when implemented in routine clinical workflows; to understand the long-term benefits and potential limitations of using the predictive model; to refine the model based on real-world data and feedback.

## Conclusion

In summary, our study highlights the importance of CRGs in the etiology of osteoporosis and offers fresh insights into the genetic foundations of the condition. Our findings highlight the potential of personalized medicine in osteoporosis treatment by the correlation found between CRGs and immune cell infiltration, the identification of unique clusters, and the creation of a prediction model. While our model identifies potential biomarkers, future research should investigate the therapeutic potential of targeting CRGs in clinical settings and try to validate the model in larger, multi-ethnic cohorts with standardized phenotyping to mitigate confounding effects.

## Data Availability

The original contributions presented in the study are included in the article/Supplementary Material, further inquiries can be directed to the corresponding author.

## References

[B1] Absar N. Mamur B. Mahmud A. Emran T. B. Khandaker M. U. Faruque M. R. I. (2022). Development of a computer-aided tool for detection of COVID-19 pneumonia from CXR images using machine learning algorithm. J. Radiat. Res. Appl. Sci. 15 (1), 32–43. 10.1016/j.jrras.2022.02.002

[B2] Baert L. (1974). Controlled double-blind trail of flavoxate in painful conditions of the lower urinary tract. Curr. Med. Res. Opin. 2 (10), 631–635. 10.1185/03007997409111875 4616803

[B3] Bates M. E. Troop L. Brown M. E. Puetzer J. L. (2023). Temporal application of lysyl oxidase during hierarchical collagen fiber formation differentially effects tissue mechanics. Acta Biomater. 160, 98–111. 10.1016/j.actbio.2023.02.024 36822485 PMC10064799

[B4] Benisch P. Schilling T. Klein-Hitpass L. Frey S. P. Seefried L. Raaijmakers N. (2012). The transcriptional profile of mesenchymal stem cell populations in primary osteoporosis is distinct and shows overexpression of osteogenic inhibitors. PLoS One 7 (9), e45142. 10.1371/journal.pone.0045142 23028809 PMC3454401

[B5] Bereket C. Sener I. Cakir-Ozkan N. Onger M. E. Polat A. V. (2018). Beneficial therapeutic effects of sildenafil on bone healing in animals treated with bisphosphonate. Niger. J. Clin. Pract. 21 (2), 217–224. 10.4103/njcp.njcp_172_16 29465058

[B6] Cao S. Wang Q. Sun Z. Zhang Y. Liu Q. Huang Q. (2023). Role of cuproptosis in understanding diseases. Hum. Cell 36 (4), 1244–1252. 10.1007/s13577-023-00914-6 37154876 PMC10165592

[B7] Chaudhri M. A. Kemmler W. Harsch I. Watling R. J. (2009). Plasma copper and bone mineral density in osteopenia: an indicator of bone mineral density in osteopenic females. Biol. Trace Elem. Res. 129 (1-3), 94–98. 10.1007/s12011-008-8299-0 19139831

[B8] Chen X. Lan X. Mo S. Qin J. Li W. Liu P. (2009). p38 and ERK, but not JNK, are involved in copper-induced apoptosis in cultured cerebellar granule neurons. Biochem. Biophys. Res. Commun. 379 (4), 944–948. 10.1016/j.bbrc.2008.12.177 19138669

[B9] Chen Q. Liu K. Robinson A. R. Clauson C. L. Blair H. C. Robbins P. D. (2013). DNA damage drives accelerated bone aging via an NF-κB-dependent mechanism. J. Bone Min. Res. 28 (5), 1214–1228. 10.1002/jbmr.1851 23281008 PMC3662975

[B10] Chen G. Wang C. Wang J. Yin S. Gao H. Xiang L. U. (2016). Antiosteoporotic effect of icariin in ovariectomized rats is mediated via the Wnt/β-catenin pathway. Exp. Ther. Med. 12 (1), 279–287. 10.3892/etm.2016.3333 27347050 PMC4906828

[B11] Chen B. Khodadoust M. S. Liu C. L. Newman A. M. Alizadeh A. A. (2018). Profiling tumor infiltrating immune cells with CIBERSORT. Methods Mol. Biol. 1711, 243–259. 10.1007/978-1-4939-7493-1_12 29344893 PMC5895181

[B12] Chen X. Cai Q. Liang R. Zhang D. Liu X. Zhang M. (2023). Copper homeostasis and copper-induced cell death in the pathogenesis of cardiovascular disease and therapeutic strategies. Cell Death Dis. 14 (2), 105. 10.1038/s41419-023-05639-w 36774340 PMC9922317

[B13] Chiu C. C. Weng Y. H. Huang Y. Z. Chen R. S. Liu Y. C. Yeh T. H. (2020). (D620N) VPS35 causes the impairment of Wnt/β-catenin signaling cascade and mitochondrial dysfunction in a PARK17 knockin mouse model. Cell Death Dis. 11 (11), 1018. 10.1038/s41419-020-03228-9 33257649 PMC7705022

[B14] Curtis E. M. Murray R. Titcombe P. Cook E. Clarke-Harris R. Costello P. (2017). Perinatal DNA methylation at CDKN2A is associated with Offspring bone mass: findings from the Southampton Women's Survey. J. Bone Min. Res. 32 (10), 2030–2040. 10.1002/jbmr.3153 28419547 PMC5528139

[B15] Curtis E. M. Woolford S. Holmes C. Cooper C. Harvey N. C. (2020). General and specific Considerations as to why osteoporosis-related Care is Often Suboptimal. Curr. Osteoporos. Rep. 18 (1), 38–46. 10.1007/s11914-020-00566-7 32103393 PMC7067731

[B16] Dong J. Wang X. Xu C. Gao M. Wang S. Zhang J. (2021). Inhibiting NLRP3 inflammasome activation prevents copper-induced neuropathology in a murine model of Wilson's disease. Cell Death Dis. 12 (1), 87. 10.1038/s41419-021-03397-1 33462188 PMC7813851

[B17] Ensrud K. E. Crandall C. J. (2024). Osteoporosis. Ann. Intern Med. 177 (1), ITC1–ITC16. 10.7326/AITC202401160 38190715

[B18] Epstein P. M. Basole C. Brocke S. (2021). The role of PDE8 in T cell Recruitment and function in inflammation. Front. Cell Dev. Biol. 9, 636778. 10.3389/fcell.2021.636778 33937235 PMC8085600

[B19] Evangelista J. E. Xie Z. Marino G. B. Nguyen N. Clarke D. J. B. Ma'ayan A. (2023). Enrichr-KG: bridging enrichment analysis across multiple libraries. Nucleic Acids Res. 51 (W1), W168–W179. 10.1093/nar/gkad393 37166973 PMC10320098

[B20] Fan Y. Ni S. Zhang H. (2022). Associations of copper Intake with bone mineral density and osteoporosis in adults: data from the National health and Nutrition Examination Survey. Biol. Trace Elem. Res. 200 (5), 2062–2068. 10.1007/s12011-021-02845-5 34283365

[B21] Florencio-Silva R. Sasso G. R. Sasso-Cerri E. Simoes M. J. Cerri P. S. (2015). Biology of bone tissue: structure, function, and factors that Influence bone cells. Biomed. Res. Int. 2015, 421746. 10.1155/2015/421746 26247020 PMC4515490

[B22] Frase D. Lee C. Nachiappan C. Gupta R. Akkouch A. (2023). The inflammatory contribution of B-lymphocytes and neutrophils in progression to osteoporosis. Cells 12 (13), 1744. 10.3390/cells12131744 37443778 PMC10340451

[B23] Gao Y. Chen N. Fu Z. Zhang Q. (2023). Progress of Wnt signaling pathway in osteoporosis. Biomolecules 13 (3), 483. 10.3390/biom13030483 36979418 PMC10046187

[B24] Gao P. Pan X. Wang S. Guo S. Dong Z. Wang Z. (2024). Identification of the transcriptome signatures and immune-inflammatory responses in postmenopausal osteoporosis. Heliyon 10 (1), e23675. 10.1016/j.heliyon.2023.e23675 38187229 PMC10770509

[B25] Ge E. J. Bush A. I. Casini A. Cobine P. A. Cross J. R. DeNicola G. M. (2022). Connecting copper and cancer: from transition metal signalling to metalloplasia. Nat. Rev. Cancer 22 (2), 102–113. 10.1038/s41568-021-00417-2 34764459 PMC8810673

[B26] Gillespie M. Jassal B. Stephan R. Milacic M. Rothfels K. Senff-Ribeiro A. (2022). The reactome pathway knowledgebase 2022. Nucleic Acids Res. 50 (D1), D687–D692. 10.1093/nar/gkab1028 34788843 PMC8689983

[B27] Han X. F. Zhang X. X. Liu K. M. Zhang Q. (2018). Apelin-13 deficiency alters cortical bone geometry, organic bone matrix, and inhibits Wnt/β-catenin signaling. Gen. Comp. Endocrinol. 267, 29–35. 10.1016/j.ygcen.2018.05.024 29857005

[B28] Hayes M. T. (2019). Parkinson's disease and Parkinsonism. Am. J. Med. 132 (7), 802–807. 10.1016/j.amjmed.2019.03.001 30890425

[B29] He J. Li W. Zhao W. Shen H. Chang Y. Liu B. (2024). Potential of lncRNAs to regulate cuproptosis in hepatocellular carcinoma: Establishment and validation of a novel risk model. Heliyon 10 (2), e24453. 10.1016/j.heliyon.2024.e24453 38312553 PMC10835266

[B30] Henriksen C. Arnesen E. K. (2023). Copper - a scoping review for Nordic Nutrition Recommendations 2023. Food Nutr. Res. 67. 10.29219/fnr.v67.10322 38084148 PMC10710866

[B31] Huang Y. Yin D. Wu L. (2022). Identification of cuproptosis-related subtypes and development of a prognostic signature in colorectal cancer. Sci. Rep. 12 (1), 17348. 10.1038/s41598-022-22300-2 36253436 PMC9576756

[B32] Jakovljevic A. Nikolic N. Paterno Holtzman L. Tournier P. Gaudin A. Cordaro L. (2023). Involvement of the Notch signaling system in alveolar bone resorption. Jpn. Dent. Sci. Rev. 59, 38–47. 10.1016/j.jdsr.2023.02.003 36880060 PMC9985033

[B33] Ji J. Wu S. Bao X. Liu S. Ye Y. Liu J. (2023). Mediating oxidative stress through the Palbociclib/miR-141-3p/STAT4 axis in osteoporosis: a bioinformatics and experimental validation study. Sci. Rep. 13 (1), 19560. 10.1038/s41598-023-46813-6 37949959 PMC10638393

[B34] Jiang M. Liu K. Lu S. Qiu Y. Zou X. Zhang K. (2023). Verification of cuproptosis-related diagnostic model associated with immune infiltration in rheumatoid arthritis. Front. Endocrinol. (Lausanne) 14, 1204926. 10.3389/fendo.2023.1204926 37547319 PMC10399571

[B35] Jiao Y. Feng Y. Wang X. (2018). Regulation of Tumor suppressor gene CDKN2A and Encoded p16-INK4a protein by Covalent Modifications. Biochem. (Mosc) 83 (11), 1289–1298. 10.1134/S0006297918110019 30482142

[B36] Jilka R. L. Noble B. Weinstein R. S. (2013). Osteocyte apoptosis. Bone 54 (2), 264–271. 10.1016/j.bone.2012.11.038 23238124 PMC3624050

[B37] Kanehisa M. Furumichi M. Tanabe M. Sato Y. Morishima K. (2017). KEGG: new perspectives on genomes, pathways, diseases and drugs. Nucleic Acids Res. 45 (D1), D353-D361–D361. 10.1093/nar/gkw1092 27899662 PMC5210567

[B38] Kang Y. J. Yoo J. I. Baek K. W. (2021). Differential gene expression profile by RNA sequencing study of elderly osteoporotic hip fracture patients with sarcopenia. J. Orthop. Transl. 29, 10–18. 10.1016/j.jot.2021.04.009 34036042 PMC8138673

[B39] Karakas E. Y. Yetisgin A. Cadirci D. Sezen H. Altunbas R. Kas F. (2016). Usefulness of ceruloplasmin testing as a screening methodology for geriatric patients with osteoporosis. J. Phys. Ther. Sci. 28 (1), 235–239. 10.1589/jpts.28.235 26957765 PMC4756011

[B40] Keum B. R. Kim H. J. Lee J. Lee M. Hong S. H. Chang H. K. (2024). Heterogeneous osteoimmune profiles via single-cell transcriptomics in osteoporotic patients who fail bisphosphonate treatment. Proc. Natl. Acad. Sci. U. S. A. 121 (8), e2316871121. 10.1073/pnas.2316871121 38346184 PMC10895260

[B41] Kucukcongar Yavas A. Basan H. Dincer S. Bilginer Gurbuz B. Kasapkara C. S. (2024). Mitochondrial phosphate-carrier deficiency mimicking infantile-onset Pompe disease. Am. J. Med. Genet. A 194, e63643. 10.1002/ajmg.a.63643 38656665

[B42] Li Y. Xie B. Jiang Z. Yuan B. (2019). Relationship between osteoporosis and osteoarthritis based on DNA methylation. Int. J. Clin. Exp. Pathol. 12 (9), 3399–3407. 31934183 PMC6949860

[B43] Li J. Wu F. Li C. Sun S. Feng C. Wu H. (2022). The cuproptosis-related signature predicts prognosis and indicates immune microenvironment in breast cancer. Front. Genet. 13, 977322. 10.3389/fgene.2022.977322 36226193 PMC9548612

[B44] Li D. Gao Z. Li Q. Liu X. Liu H. (2023). Cuproptosis-a potential target for the treatment of osteoporosis. Front. Endocrinol. (Lausanne) 14, 1135181. 10.3389/fendo.2023.1135181 37214253 PMC10196240

[B45] Liu H. (2022). Pan-cancer profiles of the cuproptosis gene set. Am. J. Cancer Res. 12 (8), 4074–4081. 36119826 PMC9442004

[B46] Liu N. Chen M. (2024). Crosstalk between ferroptosis and cuproptosis: from mechanism to potential clinical application. Biomed. Pharmacother. 171, 116115. 10.1016/j.biopha.2023.116115 38181713

[B47] Liu H. Xiong Y. Zhu X. Gao H. Yin S. Wang J. (2017). Icariin improves osteoporosis, inhibits the expression of PPARγ, C/EBPα, FABP4 mRNA, N1ICD and jagged1 proteins, and increases Notch2 mRNA in ovariectomized rats. Exp. Ther. Med. 13 (4), 1360–1368. 10.3892/etm.2017.4128 28413478 PMC5377361

[B48] Liu Y. Zhu J. Xu L. Wang B. Lin W. Luo Y. (2022a). Copper regulation of immune response and potential implications for treating orthopedic disorders. Front. Mol. Biosci. 9, 1065265. 10.3389/fmolb.2022.1065265 36545506 PMC9762617

[B49] Liu Z. Wang L. Xing Q. Liu X. Hu Y. Li W. (2022b). Identification of GLS as a cuproptosis-related diagnosis gene in acute myocardial infarction. Front. Cardiovasc Med. 9, 1016081. 10.3389/fcvm.2022.1016081 36440046 PMC9691691

[B50] Liu M. Li Y. Han S. Wang H. Li J. (2023a). Activin A alleviates neuronal injury through inhibiting cGAS-STING-mediated autophagy in mice with ischemic stroke. J. Cereb. Blood Flow. Metab. 43 (5), 736–748. 10.1177/0271678X221147056 36537048 PMC10108189

[B51] Liu S. Ge J. Chu Y. Cai S. Wu J. Gong A. (2023b). Identification of hub cuproptosis related genes and immune cell infiltration characteristics in periodontitis. Front. Immunol. 14, 1164667. 10.3389/fimmu.2023.1164667 37215133 PMC10196202

[B52] Liu J. Lustberg D. J. Galvez A. Liles L. C. McCann K. E. Weinshenker D. (2024). Genetic disruption of dopamine beta-hydroxylase dysregulates innate responses to predator odor in mice. Neurobiol. Stress 29, 100612. 10.1016/j.ynstr.2024.100612 38371489 PMC10873756

[B53] Macatangay B. J. C. Jackson E. K. Abebe K. Z. Comer D. Cyktor J. Klamar-Blain C. (2020). A Randomized, Placebo-Controlled, Pilot clinical trial of dipyridamole to decrease human Immunodeficiency Virus-associated chronic inflammation. J. Infect. Dis. 221 (10), 1598–1606. 10.1093/infdis/jiz344 31282542 PMC7184919

[B54] Mahdavi-Roshan M. Ebrahimi M. Ebrahimi A. (2015). Copper, magnesium, zinc and calcium status in osteopenic and osteoporotic post-menopausal women. Clin. Cases Min. Bone Metab. 12 (1), 18–21. 10.11138/ccmbm/2015.12.1.018 26136790 PMC4469220

[B55] Mao Y. Xia T. Hu F. Chen D. He Y. Bi X. (2024). The greener the living environment, the better the health? Examining the effects of multiple green exposure metrics on physical activity and health among young students. Environ. Res. 250, 118520. 10.1016/j.envres.2024.118520 38401683

[B56] Marini F. Giusti F. Palmini G. Brandi M. L. (2023). Role of Wnt signaling and sclerostin in bone and as therapeutic targets in skeletal disorders. Osteoporos. Int. 34 (2), 213–238. 10.1007/s00198-022-06523-7 35982318

[B57] Martens M. Ammar A. Riutta A. Waagmeester A. Slenter D. N. Hanspers K. (2021). WikiPathways: connecting communities. Nucleic Acids Res. 49 (D1), D613–D621. 10.1093/nar/gkaa1024 33211851 PMC7779061

[B58] Mir E. Arash H.-n. Amir B. E J. Bekheirnia M. A Afshar N. (2007). Adequate serum copper concentration could improve bone density, postpone bone loss and protect osteoporosis in women. Iran. J. Public Health, 24–29.

[B59] Mollazadeh S. Fazly Bazzaz B. S. Kerachian M. A. (2015). Role of apoptosis in pathogenesis and treatment of bone-related diseases. J. Orthop. Surg. Res. 10, 15. 10.1186/s13018-015-0152-5 25627748 PMC4327805

[B60] Newman A. M. Steen C. B. Liu C. L. Gentles A. J. Chaudhuri A. A. Scherer F. (2019). Determining cell type abundance and expression from bulk tissues with digital cytometry. Nat. Biotechnol. 37 (7), 773–782. 10.1038/s41587-019-0114-2 31061481 PMC6610714

[B61] Nguyen H. D. Nguyen Q. H. Dang D. K. Van C. P. Truong Q. H. Pham S. D. (2024). A novel flood risk management approach based on future climate and land use change scenarios. Sci. Total Environ. 921, 171204. 10.1016/j.scitotenv.2024.171204 38401735

[B62] Pomaznoy M. Ha B. Peters B. (2018). GOnet: a tool for interactive Gene Ontology analysis. BMC Bioinforma. 19 (1), 470. 10.1186/s12859-018-2533-3 30526489 PMC6286514

[B63] Qiao L. Zhu G. Jiang T. Qian Y. Sun Q. Zhao G. (2024). Self-destructive copper Carriers Induce pyroptosis and cuproptosis for Efficient Tumor Immunotherapy against Dormant and Recurrent Tumors. Adv. Mater 36 (8), e2308241. 10.1002/adma.202308241 37820717

[B64] Qu X. He Z. Qiao H. Zhai Z. Mao Z. Yu Z. (2018). Serum copper levels are associated with bone mineral density and total fracture. J. Orthop. Transl. 14, 34–44. 10.1016/j.jot.2018.05.001 30035031 PMC6034109

[B65] Sarafrazi N. Wambogo E. A. Shepherd J. A. (2021). Osteoporosis or low bone mass in Older Adults: United States, 2017–2018. NCHS data brief. 405, 1–8. 34029181

[B66] Shao J. L. Li H. Zhang X. R. Zhang X. Li Z. Z. Jiao G. L. (2020). Identification of serum Exosomal MicroRNA expression profiling in menopausal females with osteoporosis by high-throughput sequencing. Curr. Med. Sci. 40 (6), 1161–1169. 10.1007/s11596-020-2306-x 33428145

[B67] Singer A. J. (2021). “Chapter 29 - Economics of osteoporosis,” in Marcus and Feldman's osteoporosis. Fifth Edition (Academic Press), 693–704.

[B68] Stevenson J. medical advisory council of the British Menopause, S (2023). Prevention and treatment of osteoporosis in women. Post. Reprod. Health 29 (1), 11–14. 10.1177/20533691221139902 36357006 PMC10009319

[B69] Tsvetkov P. Coy S. Petrova B. Dreishpoon M. Verma A. Abdusamad M. (2022). Copper induces cell death by targeting lipoylated TCA cycle proteins. Science 375 (6586), 1254–1261. 10.1126/science.abf0529 35298263 PMC9273333

[B70] Turnlund J. R. Keyes W. R. Peiffer G. L. Scott K. C. (1998). Copper absorption, excretion, and retention by young men consuming low dietary copper determined by using the stable isotope 65Cu. Am. J. Clin. Nutr. 67 (6), 1219–1225. 10.1093/ajcn/67.6.1219 9625096

[B71] Wang H. Du D. Huang J. Wang S. He X. Yuan S. (2022a). GPR27 regulates hepatocellular carcinoma progression via MAPK/ERK pathway. Cancer Manag. Res. 14, 1165–1177. 10.2147/CMAR.S335749 35330739 PMC8938170

[B72] Wang X. Zhang X. Han Y. Duan X. Wang J. Yan H. (2022b). Role of the major histocompatibility complex class II protein presentation pathway in bone immunity imbalance in postmenopausal osteoporosis. Front. Endocrinol. (Lausanne) 13, 876067. 10.3389/fendo.2022.876067 36034452 PMC9402988

[B73] Wang Y. Qiu X. Liu J. Liu X. Pan J. Cai J. (2024). Cuproptosis-related biomarkers and Characterization of immune infiltration in sepsis. J. Inflamm. Res. 17, 2459–2478. 10.2147/JIR.S452980 38681070 PMC11048236

[B74] Warjukar P. R. Paunipagar R. P. Timalsina D. R. Mohabey A. V. Jain P. B. Panbude S. P. (2024). Ceruloplasmin, vitamin C, and Uric acid levels in patients with myocardial infarction: a comparative cross-Sectional study. Cureus 16 (3), e56122. 10.7759/cureus.56122 38618322 PMC11015052

[B75] Weinstein R. S. Manolagas S. C. (2000). Apoptosis and osteoporosis. Am. J. Med. 108 (2), 153–164. 10.1016/s0002-9343(99)00420-9 11126309

[B76] Xia W. F. Tang F. L. Xiong L. Xiong S. Jung J. U. Lee D. H. (2013). Vps35 loss promotes hyperresorptive osteoclastogenesis and osteoporosis via sustained RANKL signaling. J. Cell Biol. 200 (6), 821–837. 10.1083/jcb.201207154 23509071 PMC3601351

[B77] Xiao P. Chen Y. Jiang H. Liu Y. Z. Pan F. Yang T. L. (2008). *In vivo* genome-wide expression study on human circulating B cells suggests a novel ESR1 and MAPK3 network for postmenopausal osteoporosis. J. Bone Min. Res. 23 (5), 644–654. 10.1359/jbmr.080105 18433299 PMC2674539

[B78] Xu J. Hu Z. Cao H. Zhang H. Luo P. Zhang J. (2022). Multi-omics pan-cancer study of cuproptosis core gene FDX1 and its role in kidney renal clear cell carcinoma. Front. Immunol. 13, 981764. 10.3389/fimmu.2022.981764 36605188 PMC9810262

[B79] Xu J. Song Y. Ding S. Duan W. Xiang G. Wang Z. (2024). Myeloid-derived growth factor and its effects on cardiovascular and metabolic diseases. Cytokine Growth Factor Rev. 76, 77–85. 10.1016/j.cytogfr.2023.12.005 38185568

[B80] Xue Q. Kang R. Klionsky D. J. Tang D. Liu J. Chen X. (2023). Copper metabolism in cell death and autophagy. Autophagy 19 (8), 2175–2195. 10.1080/15548627.2023.2200554 37055935 PMC10351475

[B81] Xue P. Sanchez-Leon E. Hu G. Lee C. W. Black B. Brisland A. (2024). The interplay between electron transport chain function and iron regulatory factors influences melanin formation in Cryptococcus neoformans. bioRxiv, 2024.02.15.580540. 10.1101/2024.02.15.580540 38687055 PMC11237718

[B82] Yalcin Z. Koot D. Bezstarosti K. Salas-Lloret D. Bleijerveld O. B. Boersma V. (2023). Ubiquitinome profiling reveals *in vivo* UBE2D3 targets and Implicates UBE2D3 in protein quality control. Mol. Cell Proteomics 22 (6), 100548. 10.1016/j.mcpro.2023.100548 37059365 PMC10209342

[B83] Yan C. Niu Y. Ma L. Tian L. Ma J. (2022). System analysis based on the cuproptosis-related genes identifies LIPT1 as a novel therapy target for liver hepatocellular carcinoma. J. Transl. Med. 20 (1), 452. 10.1186/s12967-022-03630-1 36195876 PMC9531858

[B84] Yang L. Jia X. Fu Y. Tian J. Liu Y. Lin J. (2023a). Creation of a prognostic model using cuproptosis-associated long Noncoding RNAs in hepatocellular carcinoma. Int. J. Mol. Sci. 24 (12), 9987. 10.3390/ijms24129987 37373132 PMC10298112

[B85] Yang W. Wang Y. Huang Y. Yu J. Wang T. Li C. (2023b). 4-Octyl itaconate inhibits aerobic glycolysis by targeting GAPDH to promote cuproptosis in colorectal cancer. Biomed. Pharmacother. 159, 114301. 10.1016/j.biopha.2023.114301 36706634

[B86] Yang M. Wang Y. He L. Shi X. Huang S. (2024). Comprehensive bioinformatics analysis reveals the role of cuproptosis-related gene Ube2d3 in myocardial infarction. Front. Immunol. 15, 1353111. 10.3389/fimmu.2024.1353111 38440726 PMC10909922

[B87] Yoshida G. Kawabata T. Takamatsu H. Saita S. Nakamura S. Nishikawa K. (2022). Degradation of the NOTCH intracellular domain by elevated autophagy in osteoblasts promotes osteoblast differentiation and alleviates osteoporosis. Autophagy 18 (10), 2323–2332. 10.1080/15548627.2021.2017587 35025696 PMC9542956

[B88] Yu W. Liu H. Zhang Y. Liu M. Li W. Wang L. (2024). Identification of 10 differentially expressed and cuproptosis-related genes in immune infiltration and prognosis of thyroid carcinoma. Cell Mol. Biol. (Noisy-le-grand) 70 (3), 89–94. 10.14715/cmb/2024.70.3.13 38650151

[B89] Yuan D. Li X. Q. Qu F. W. Wang Y. (2023). Landscape and the immune patterns of cuproptosis in oral squamous cell carcinoma. J. Oral Pathol. Med. 52 (10), 951–960. 10.1111/jop.13489 37828627

[B90] Zaidi M. Kim S. M. Mathew M. Korkmaz F. Sultana F. Miyashita S. (2023). Bone circuitry and interorgan skeletal crosstalk. Elife 12, e83142. 10.7554/eLife.83142 36656634 PMC9851618

[B91] Zhang B. Burke R. (2023). Copper homeostasis and the ubiquitin proteasome system. Metallomics 15 (3), mfad010. 10.1093/mtomcs/mfad010 36822629 PMC10022722

[B92] Zhang G. Sun J. Zhang X. (2022). A novel Cuproptosis-related LncRNA signature to predict prognosis in hepatocellular carcinoma. Sci. Rep. 12 (1), 11325. 10.1038/s41598-022-15251-1 35790864 PMC9256635

[B93] Zhou Y. Gao Y. Xu C. Shen H. Tian Q. Deng H. W. (2018). A novel approach for correction of crosstalk effects in pathway analysis and its application in osteoporosis research. Sci. Rep. 8 (1), 668. 10.1038/s41598-018-19196-2 29330445 PMC5766601

[B94] Zhu X. Chen H. Li H. Ren H. Ye C. Xu K. (2023a). ITGB1-mediated molecular landscape and cuproptosis phenotype induced the worse prognosis in diffuse gastric cancer. Front. Oncol. 13, 1115510. 10.3389/fonc.2023.1115510 37007126 PMC10063208

[B95] Zhu Y. Chang S. Liu J. Wang B. (2023b). Identification of a novel cuproptosis-related gene signature for multiple myeloma diagnosis. Immun. Inflamm. Dis. 11 (11), e1058. 10.1002/iid3.1058 38018590 PMC10629272

