# Supplementary figures and images for: Cuproptosis related genes in immune infiltration and treatment of osteoporosis by bioinformatic analysis and machine learning methods
Source: Front Physiol. 2025 Sep 4;16:1605473. doi: 10.3389/fphys.2025.1605473 (PMC12443804; doi:10.3389/fphys.2025.1605473)

Network heatmap plot, selected genes

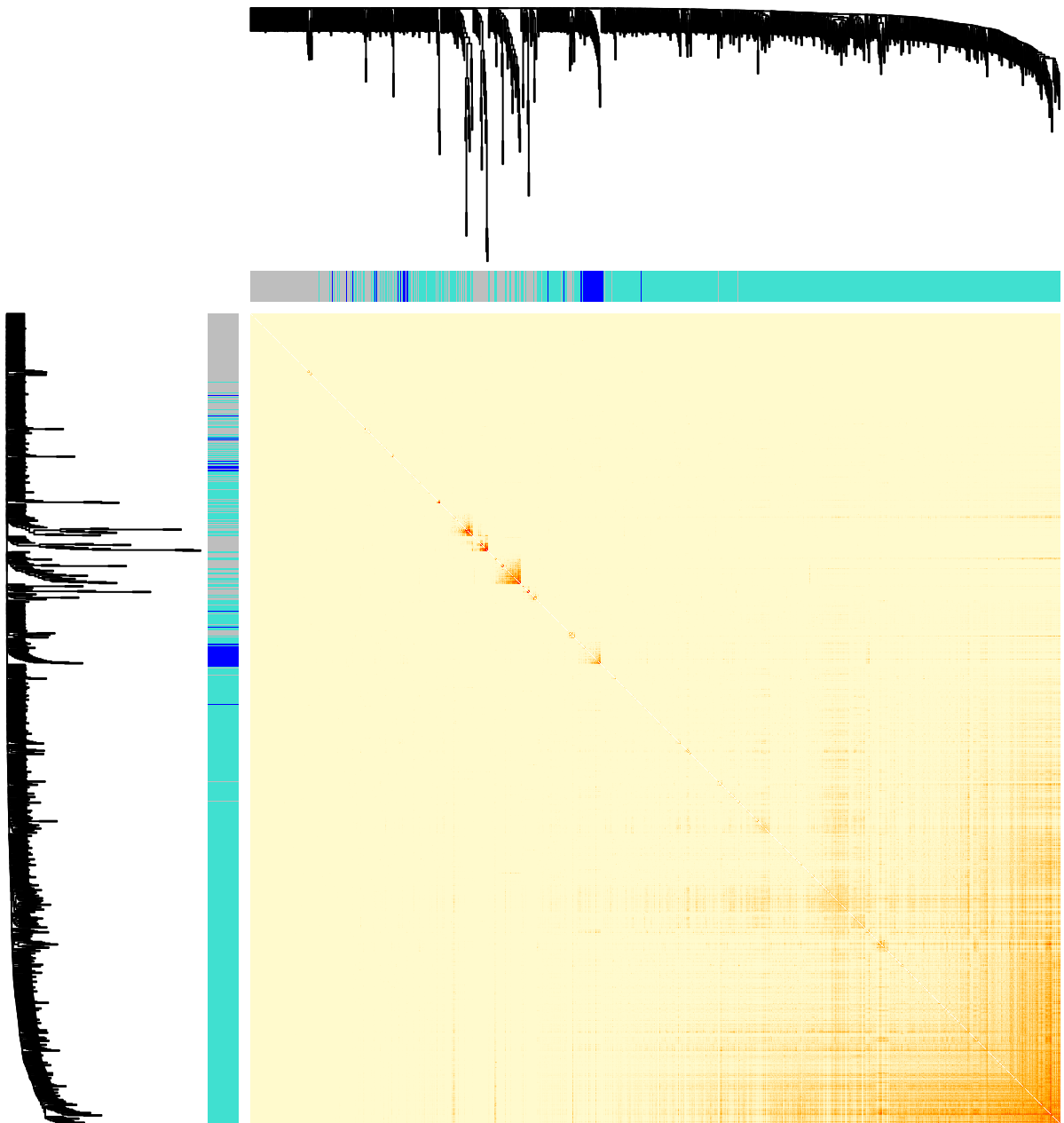

Supplement: Supplementary file 1 [file DataSheet2.pdf]

Network heatmap plot, selected genes

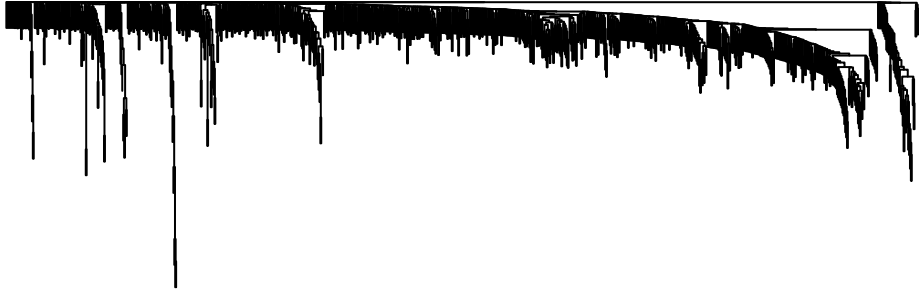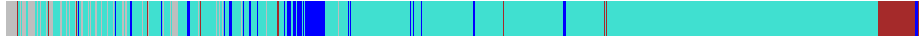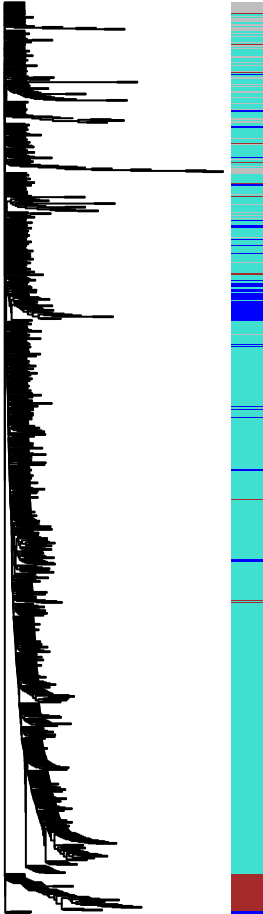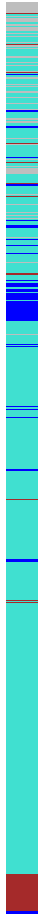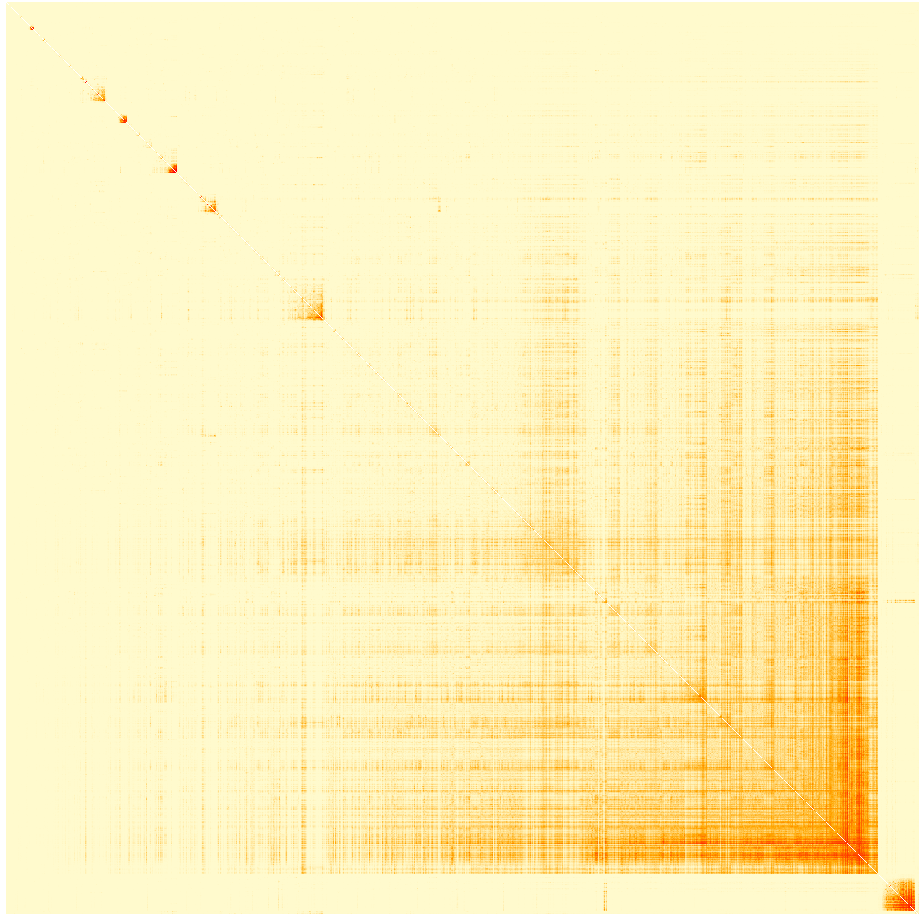

Supplement: Supplementary file 4 [file DataSheet1.pdf]

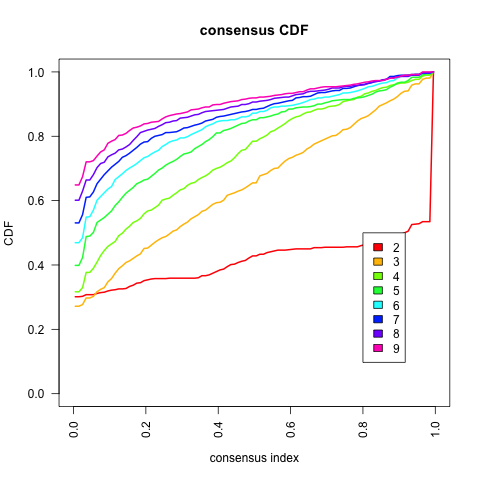

Supplement: Supplementary file 6 [file Image2.png]

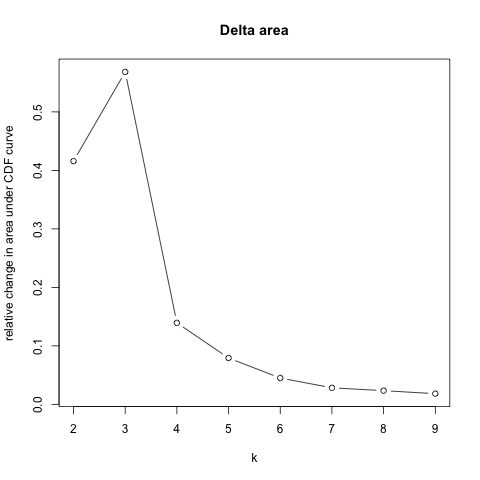

Supplement: Supplementary file 8 [file Image1.png]

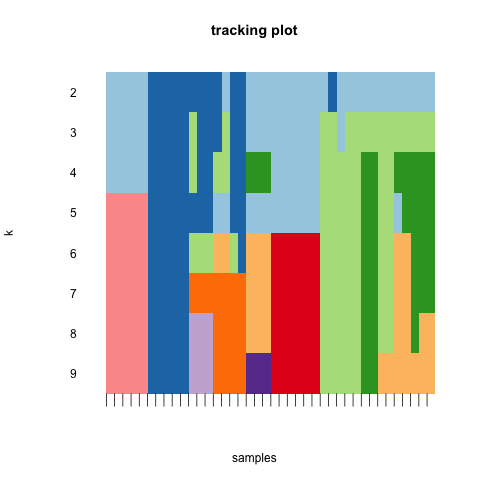

Supplement: Supplementary file 9 [file Image3.png]
